# Ibulocydine Inhibits Migration and Invasion of TNBC Cells via MMP-9 Regulation

**DOI:** 10.3390/ijms25116123

**Published:** 2024-06-01

**Authors:** Mi-Ri Kwon, Ji-Soo Park, Eun-Jung Ko, Jin Park, Eun-Jin Ju, Seol-Hwa Shin, Ga-Won Son, Hye-Won Lee, Yun-Yong Park, Myoung-Hee Kang, Yeon-Joo Kim, Byeong-Moon Kim, Hee-Jin Lee, Tae-Won Kim, Chong-Jai Kim, Si-Yeol Song, Seok-Soon Park, Seong-Yun Jeong

**Affiliations:** 1 Department of Medical Science, Asan Medical Institute of Convergence Science and Technology, University of Ulsan College of Medicine, Seoul 05505, Republic of Korea; 2 Asan Institute for Life Sciences, Asan Medical Center, Seoul 05505, Republic of Korea; 3 Asan Preclinical Evaluation Center for Cancer Therapeutix, Asan Medical Center, Seoul 05505, Republic of Korea; 4 Department of Chemistry, Seoul National University, Seoul 08826, Republic of Korea; 5 Department of Life Science, Chung-Ang University, Seoul 06974, Republic of Korea; 6 Department of Radiation Oncology, Asan Medical Center, University of Ulsan College of Medicine, Seoul 05505, Republic of Korea; 7 Department of Pathology, Asan Medical Center, University of Ulsan College of Medicine, Seoul 05505, Republic of Korea; 8 Department of Oncology, Asan Medical Center, University of Ulsan College of Medicine, Seoul 05505, Republic of Korea; 9 Department of Biochemistry and Molecular Biology, Asan Medical Center, University of Ulsan College of Medicine, Seoul 05505, Republic of Korea

**Keywords:** ibulocydine, triple-negative breast cancer, metastasis

## Abstract

Triple-negative breast cancer (TNBC) accounts for approximately 15–20% of all breast cancer types, indicating a poor survival prognosis with a more aggressive biology of metastasis to the lung and a short response duration to available therapies. Ibulocydine (IB) is a novel (cyclin-dependent kinase) CDK7/9 inhibitor prodrug displaying potent anti-cancer effects against various cancer cell types. We performed in vitro and in vivo experiments to determine whether IB inhibits metastasis and eventually overcomes the poor drug response in TNBC. The result showed that IB inhibited the growth of TNBC cells by inducing caspase-mediated apoptosis and blocking metastasis by reducing MMP-9 expression in vitro. Concurrently, in vivo experiments using the metastasis model showed that IB inhibited metastasis of MDA-MB-231-Luc cells to the lung. Collectively, these results demonstrate that IB inhibited the growth of TNBC cells and blocked metastasis by regulating MMP-9 expression, suggesting a novel therapeutic agent for metastatic TNBC.

## 1. Introduction

Triple-negative breast cancer (TNBC) refers to breast cancer that does not express the estrogen receptor, progesterone receptor, and human epidermal growth factor receptor 2 (HER2) genes [1]. It is highly aggressive, undergoing metastasis, resistant to various treatments, and has a poor overall survival rate compared to other subtypes of breast cancer cells [2,3]. Despite multiple studies, the lack of clear molecular targets for TNBC has limited the development of therapies [1,3,4,5]. Therefore, more effective treatment strategies are required.

Cyclin-dependent kinase 7 (CDK7) plays two primary roles in regulating the cell cycle and transcription factors [6]. During cell cycle progression, CDK7 activates CDK1 and CDK2 during the S/G2 phases and CDK4/6 during the G1 phase via phosphorylation [7]. During transcription, CDK7 phosphorylates serine 5 (Ser5) and Ser7 in the RNA polymerase II (Pol II)-C-terminal domain (CTD) and CDK9, which induces Ser2 phosphorylation of the Pol II CTD [6]. CDK7/9 regulate cell division, gene transcription, and other important biological processes in normal cells; however, they are overexpressed in most carcinomas [8,9,10]. Therefore, CDK7/9 represent good anti-cancer targets because of their involvement in regulating the cell cycle and transcription [11,12].

The development of therapies targeting CDK7 is ongoing for various cancers [6,13,14]. However, early CDK7 inhibitors were not initially CDK7-specific but instead acted as multi-CDK inhibitors. Albocidib (flavopiridol), an inhibitor of CDK1, 2, 4, 6, 7, and 9, was evaluated in phase I to II clinical trials for numerous cancer types [15], although it showed a limited clinical response [16,17]. Seliciclib (roscovitine), another CDK1, 2, 5, 7, and 9 inhibitor, was assessed in clinical trials for various tumor types; however, this also exhibited limited clinical activity [18,19]. Recently, various CDK7-specific inhibitors have been developed, such as BS-181 [20], ICEC0942 (CT7001; samuraciclib) [21,22,23], LY3405105 [24], LDC4297 [25], SY-1365 [26] (phase 1), THZ1 (SY-079) [27], THZ2 [28], YKL-5-124 [29], QS1189 [30], and SY-5609 [31]. Among them, SY-5609, THZ1, and THZ2 were tested preclinically against TNBC [28,31]. ICEC0942 is currently in phase I/II clinical trials for patients with breast (TNBC, HR+/HER- breast type) or prostate cancers (clinical trial ID: NCT03363893) [32]. LY3405105 is also in phase I for advanced or metastatic solid tumors (clinical trial ID: NCT03770494). SY-1365 is currently in phase I trials for advanced solid tumors, ovarian cancer, and HR+ metastatic breast cancer (clinical trial ID: NCT03134638). In addition, SY-5609 is entering phase I in select advanced solid tumors (clinical trial ID: NCT04247126). Four CDK7 inhibitors have currently been assessed in clinical trials.

Ibulocydine (IB) is a novel CDK7/9 inhibitor prodrug with an isobutyrate ester structure that has anti-cancer effects against human hepatocellular carcinoma (HCC) cells compared to normal liver cells [33]. Previously, we reported that IB sensitizes TRAIL-induced apoptosis in HCC cells [34] and sensitizes radiotherapy in lung and colon cancer cells [35]. 

Currently, CDK7/9 inhibitors are attracting attention in breast cancer [10,12,36]. In particular, CDK7/9 are highly expressed in TNBC [12,28,37,38,39,40], and their combined expression is associated with poor prognosis [28,40]. Therefore, further studies on CDK7/9 inhibitors are needed. 

For a follow-up study of IB, we explored the association of CDK7/9 expression with TNBC and evaluated whether IB has anti-cancer efficacy and can inhibit metastasis in TNBC.

## 2. Results

### 2.1. IB Has Cytotoxic Effects on TNBC Cells

**Figure 1:**
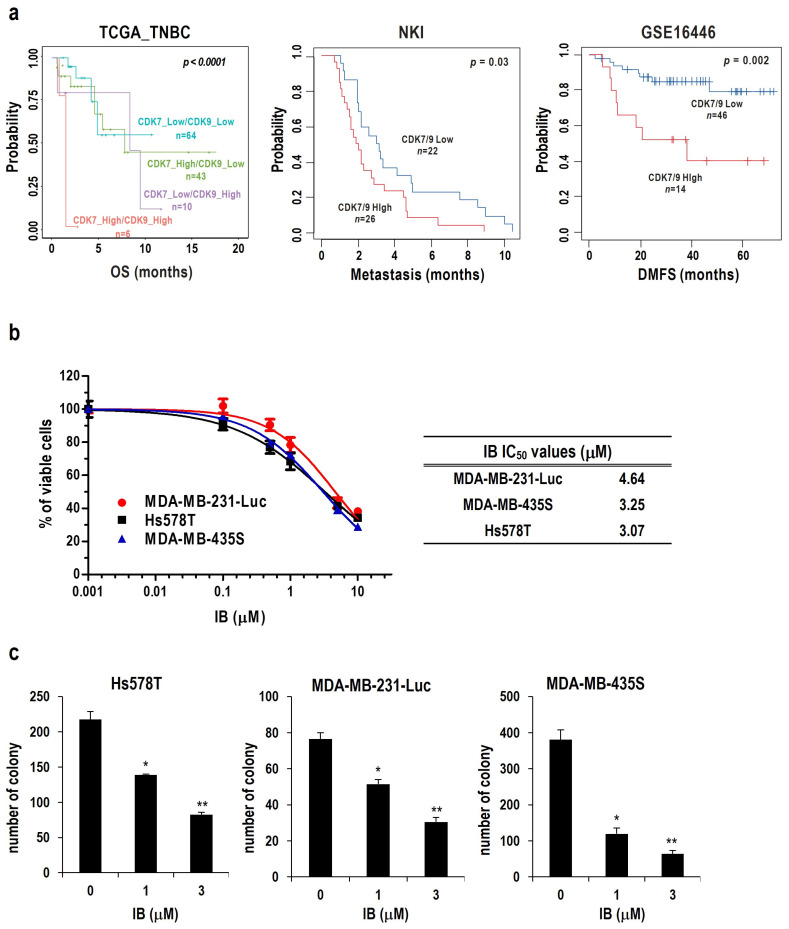
Ibulocydine induces cell death in various TNBC cells. (**a**) Patients with TNBC were divided by relatively high or low CDK7/CDK9 expression, and a Kaplan–Meier plot was generated. The differences between these groups were statistically significant regarding overall survival (OS), metastasis, and distant metastasis-free survival (DMFS). (**b**) Cell viability was measured using the CCK-8 assay after treatment with the indicated concentrations of IB for 24 h. The percentage of live cells was normalized to that of untreated control cells (100%). Data represent the mean ± SD. (**c**) Cells were treated with two different doses of IB and incubated for 10 days for colony-forming assays. Representative graphs with the number of colonies are shown. Data are presented as the mean ± SD. * *p* < 0.05, ** *p* < 0.01 vs. untreated control.

IB is an isobutyrate ester prodrug of a novel synthetic CDK inhibitor with activity against CDK7 and CDK9 [33]. To confirm the association between CDK7/9 expression and TNBC, we determined overall survival (OS) rates and CDK7/9 levels in patients with TNBC. As a result, patients with high expression of both CDK7 and CDK9 had a lower survival rate than those with high expression of CDK7 or CDK9 alone (Figure 1a; left). Furthermore, we confirmed that CDK7/9 were related not only to survival but also to metastasis. The NKI dataset showed that metastasis progressed faster in patients with high CDK7/9 expression (Figure 1a; middle). The GSE16446 dataset also revealed that patients with high CDK7/9 expression have worse outcomes regarding distant metastasis-free survival (DMFS) (Figure 1a; right). Taken together, these data demonstrated that highly expressed CDK7/9 may play a role in metastasis and survival of TNBC cells and suggest that CDK7/9 may be a good anti-cancer target for TNBC.

To examine the cytotoxic effects of IB on TNBC cells, we treated TNBC cells, including Hs578T, MDA-MB-231-Luc, and MDA-MB-435S, with different concentrations of IB for 24 h. The viability of TNBC cells after IB treatment was evaluated using a Cell Counting Kit-8 (CCK-8) assay. The results showed that IB decreased cell viability dose-dependently for various TNBC cells. The 50% inhibitory concentration (IC_50_) values for IB toward the respective cancer cell types were 4.64 μM, 3.25 μM, and 3.07 μM for MDA-MB-231-Luc, MDA-MB-435S, Hs578T, respectively (Figure 1b). In addition, IB inhibited the long-term survival of various TNBC cells in colony-forming assays (Figure 1c). 

Collectively, these results suggest that IB exerts cytotoxic effects on TNBC cells.

### 2.2. IB Induces Apoptotic Death of TNBC Cells

**Figure 2:**
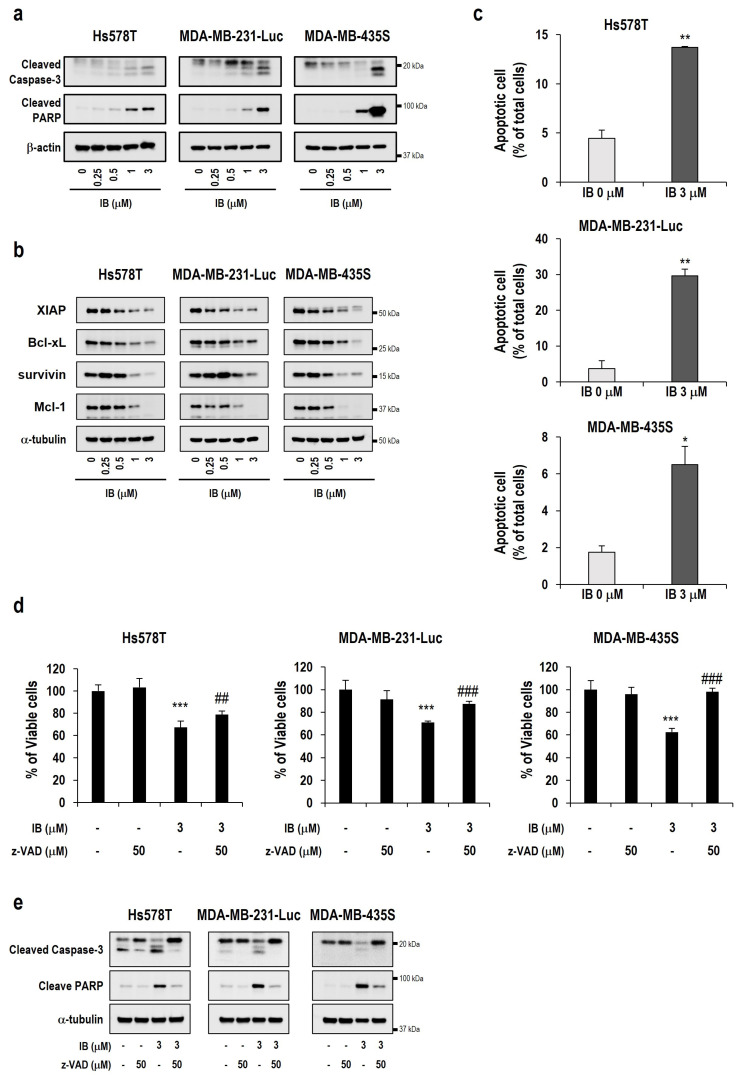
Ibulocydine induces apoptotic cell death. (**a**) TNBC cells were treated with the indicated IB concentrations for 24 h. Cell extracts were prepared from the treated cells, and Western blotting was performed using anti-cleaved caspase-3 and anti-cleaved PARP antibody. α-tubulin was used as a loading control. (**b**) Cell extracts were prepared from the cells treated with the indicated IB concentrations for 24 h. Western blotting of the anti-apoptotic family proteins was performed. α-tubulin was used as a loading control. (**c**) TNBC cells were treated with 3 μM IB for 24 h. After 24 h, apoptosis was measured with annexin V–FITC and PI staining using flow cytometry and was quantified. Data represent the mean ± SD. * *p* < 0.05, ** *p* < 0.01 vs. untreated control. (**d**) Various TNBC cells were pretreated with 50 µM z-VAD-fmk for 30 min and further treated with 3 μM IB for 24 h. Cell viability was assessed using the CCK-8 assay. Data represent the mean ± SD. *** *p* < 0.001 vs. untreated control. ## *p* < 0.01, ### *p* < 0.001 vs. IB treatment. (**e**) Cells were untreated or pretreated with 50 µM z-VAD-fmk and further treated with 3 μM IB for 24 h. Western blotting of the indicated proteins was performed. α-tubulin was used as a loading control.

To explore the mechanism of IB-induced cell death, we examined whether IB affects the expression of apoptosis-related proteins. The results showed that cleavage levels of both caspase-3 and PARP were dose-dependently increased in TNBC cells after IB treatment for 24 h (Figure 2a). In contrast, anti-apoptotic protein levels decreased after IB treatment for 24 h (Figure 2b). We further examined the effect of IB on TNBC cells by using flow cytometry after annexin V–FITC/PI staining. As a result, the percentage of annexin V positivity (+) and annexin V (+)/PI (+) increased after IB treatment in three TNBC cell lines, meaning apoptotic cell death was induced by IB (Figure 2c). Furthermore, we pretreated cells with z-VAD-fmk, a pan-caspase inhibitor, for 30 min and then treated with IB. Consequently, IB-induced cell death in TNBC cells was almost blocked by z-VAD-fmk pretreatment (Figure 2d). Additionally, IB-induced cleavage of caspase-3 and PARP was blocked by z-VAD-fmk pretreatment (Figure 2e). 

Collectively, these results demonstrate that IB induces cell death in TNBC cells via caspase-mediated apoptosis induction.

### 2.3. MMP-9 Plays a Crucial Role in IB-Induced Inhibition of Metastasis of TNBC Cells

**Figure 3:**
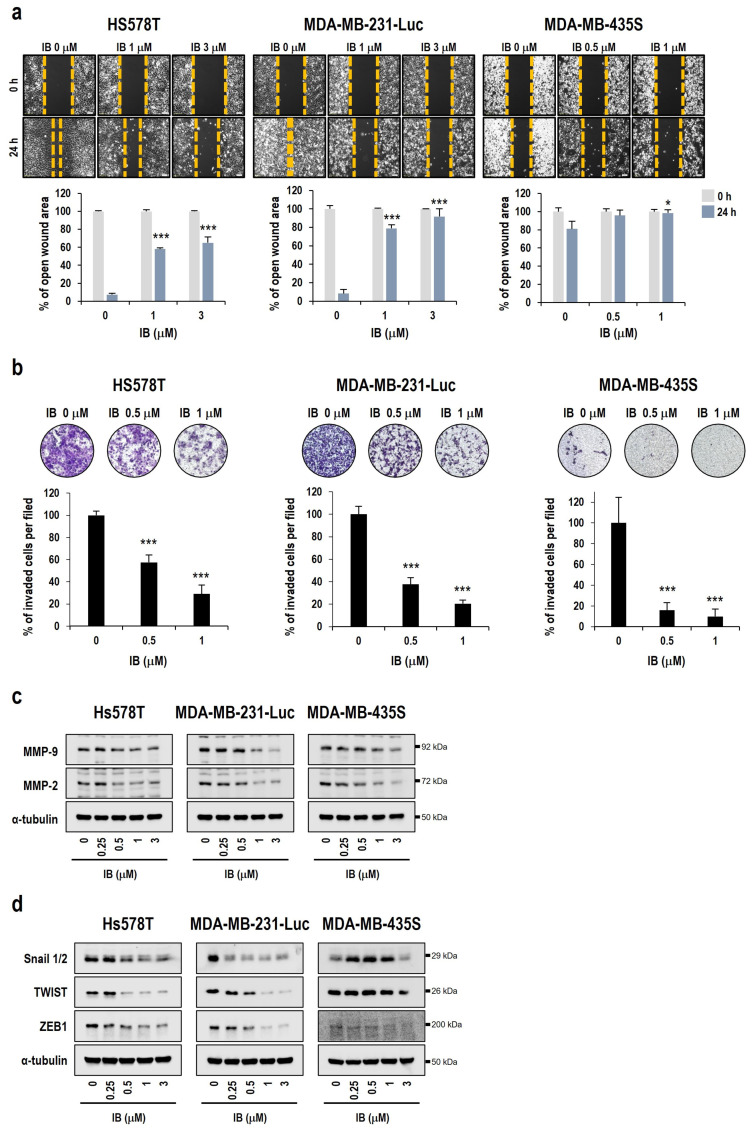

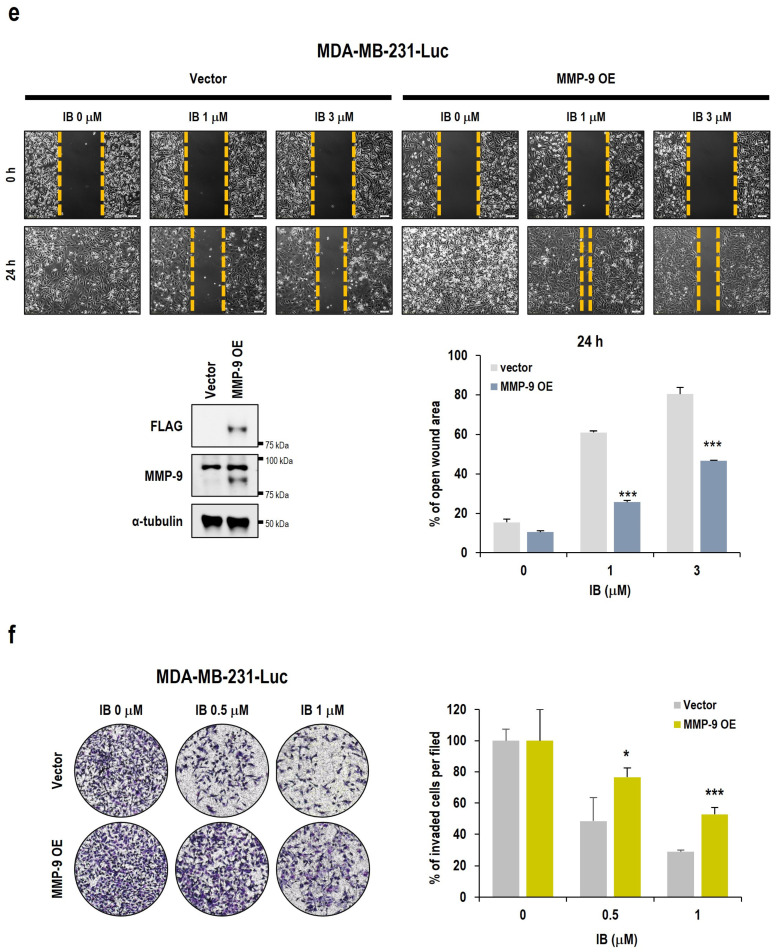
MMP-9 downregulation is critical in IB-induced metastasis inhibition in human TNBC cells. (**a**) After treatment with 1 or 3 µM IB, wound healing scratch assays were performed using TNBC cells. Cell migration was monitored under a phase-contrast microscope for 24 h: Bar, 50 μm. Data are shown as the mean ± SD. * *p* < 0.05, *** *p* < 0.001 vs. 24 h untreated control. (**b**) Invasion assays of TNBC cells treated with 0.5 or 1 µM IB for 24 h were performed. Invading cells were stained with crystal violet and observed using a fluorescence microscope (4×). Data are presented as the mean ± SD. *** *p* < 0.001 vs. untreated control. (**c**) Cell extracts were prepared from cells treated with the indicated IB concentrations for 24 h. MMP-9 and MMP-2 levels were detected using Western blotting. α-tubulin was used as a loading control. (**d**) TNBC cells were treated with the indicated IB concentrations for 24 h. Cell extracts were prepared for Western blotting of mesenchymal markers. (**e**) MMP-9 overexpressing stable cells were scratched during wound healing assays. MMP-9 upregulation was confirmed using Western blotting. Cell migration was monitored for 24 h and observed under a phase-contrast microscope: Bar, 50 μm. The graphs quantitatively show the area of wound recovery. Data are presented as the mean ± SD. *** *p* < 0.0001 vs. IB-treated vector cells. (**f**) Invasion assays of MMP-9 overexpressing stable cell lines treated with 0.5 or 1 µM IB for 24 h were performed. Invading cells were stained with crystal violet and observed using a fluorescence microscope (4×). Data are shown as the mean ± SD. * *p* < 0.05, *** *p* < 0.001 vs. IB-treated vector cells.

We evaluated whether IB inhibits the metastasis of TNBC cells because TNBC is aggressively metastatic [41]. In wound healing assays, IB effectively blocked the migration of TNBC cells into the scratch area (Figure 3a). The invasion assays also demonstrated that the number of invading TNBC cells was significantly reduced compared with that in the control cells (Figure 3b). Therefore, these results showed that IB inhibited migration and invasion of TNBC cells.

Epithelial–mesenchymal transition (EMT) is part of the metastatic process, where cancer cells lose their epithelial characteristics and gain mesenchymal characteristics [42]. To investigate the underlying mechanism in the inhibition of metastasis by IB, changes in the levels of various metastasis-regulating proteins were determined using Western blotting. The protein levels of matrix metalloproteinase-2 (MMP-2) and matrix metalloproteinase-9 (MMP-9), which are involved in the EMT and metastasis processes [43], decreased after TNBC cells were treated with IB (Figure 3c). Moreover, the levels of mesenchymal marker proteins, including Snail 1/2, TWIST, and ZEB1 [44], decreased after IB treatment (Figure 3d). Since overexpression of MMP-9 is associated with poor prognosis in patients with TNBC [45], we examined whether MMP-9 is important in the inhibition of IB-induced metastasis. For this study, we established stable MDA-MB-231-Luc cell lines with MMP-9 overexpression. MMP-9 was successfully overexpressed by the MMP-9-FLAG plasmid in MDA-MB-231-Luc cells, and its expression was confirmed using Western blotting (Figure 3e). Wound healing assay results using MMP-9 overexpressing stable cell lines showed reduced inhibition of migration by IB (Figure 3e). Likewise, the inhibition of invasion by IB was blocked in MMP-9 overexpressing cells (Figure 3f). Collectively, these results indicate that IB inhibited TNBC metastasis by downregulating MMP-9.

### 2.4. IB Inhibits Metastasis to the Lungs in an Animal Model

TNBC frequently metastasizes to the lungs [41,46,47]. Therefore, we constructed an experimental lung metastasis model using MDA-MB-231-Luc cells and evaluated the inhibition of metastasis by IB. 

**Figure 4:**
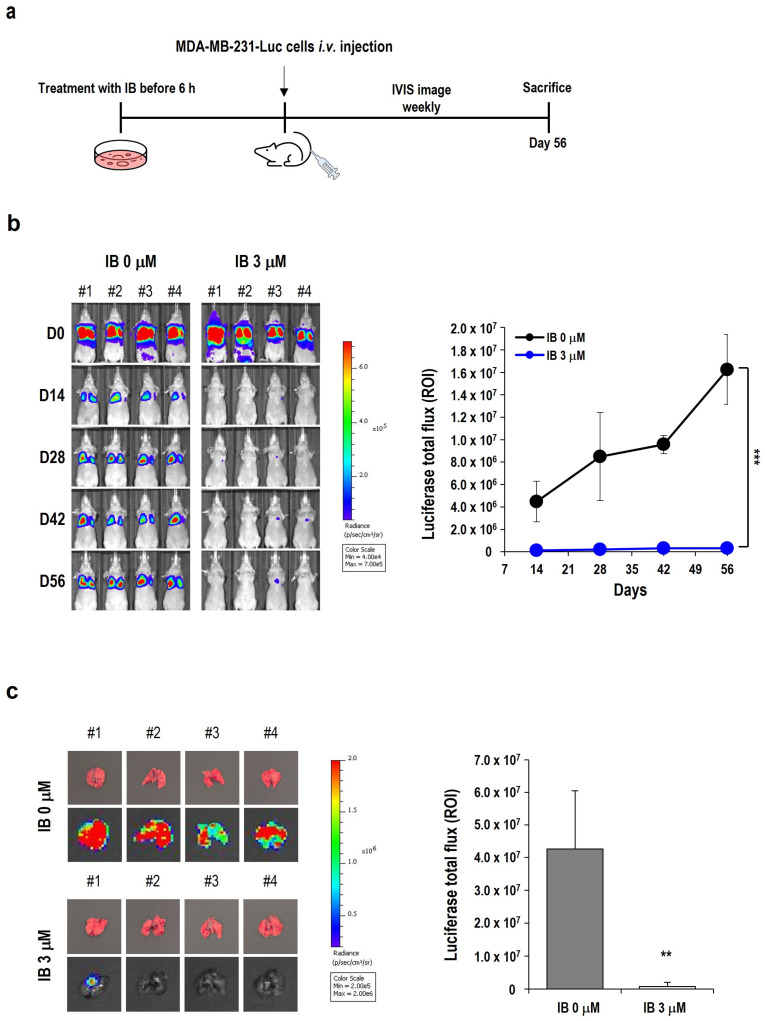
Inhibition of metastasis by IB in metastasis animal models. (**a**) Schematic diagram of the establishment of the experimental metastasis animal model and experimental schedules. (**b**) BALB/c nude mice were intravenously injected with MDA-MB-231-Luc cells treated with 3 μM IB for 6 h. For 56 days, lung metastasis was monitored by IVIS spectrum. Quantitative graphs of luciferase total flux on days 14, 28, 42, and 56 are shown. Data are presented as the mean ± SD. *** *p* < 0.001 vs. control. (**c**) Mice with lung metastasis were sacrificed at the 56-day endpoint. Lung metastasis images were obtained using the IVIS spectrum. Quantitative graphs show the luciferase total flux. Data are shown as the mean ± SD. ** *p* < 0.01 vs. control.

When we designed the experiment, we thought IB treatment after cancer cell injection would only demonstrate the anti-cancer effect. To prove that metastasis could be prevented, we planned a schedule to pretreat the cells with IB and then inject the treated cancer cells (Figure 4a). Previous results showed that treatment with 3 µM IB for 24 h showed anti-cancer effects and metastasis inhibition efficacy. We set conditions at a concentration that inhibits metastasis but does not cause cancer cell toxicity. As a result, it was confirmed that no toxicity occurred when 3 µM IB was administered for 6 h. No difference in the growth of tumor cells was observed in colony-forming assays between untreated cells and those treated with IB for 6 h (Figure S1). In the lung metastasis animal model, MDA-MB-231-Luc cells were pretreated with 3 µM IB for 6 h. After 6 h, the treated cells were injected intravenously in the tail (Figure 4a). Since intravenous injection of MDA-MB-231-Luc cells immediately goes to the lungs, luminescence activity in the lungs appears high on day 0. However, after a week, unattached cancer cells in the lungs are discharged out of the body, and the luminescence activity is lower than on the first day. As the settled cancer cells in the lungs grow, the luminescence is measured at increasingly higher levels. When the growth of metastatic tumors was monitored using the in vivo imaging system (IVIS) spectrum, tumors rarely formed in the lungs of the IB-treated group (Figure 4b). After 56 days, all mice were sacrificed and dissected to identify metastatic tumors in the lungs. IB-treated MDA-MB-231-Luc cells showed a reduction in tumor burden on the bioluminescence imaging (Figure 4c). 

These in vivo data demonstrated that IB inhibits lung metastasis in the mimic metastatic environment.

## 3. Discussion

Recently, there has been increased interest in the application of CDK inhibitors in patients with TNBC in preclinical and clinical trials [6,48,49,50]. Samuraciclib (CT7001), a CDK7 inhibitor, has completed phase 1 clinical trials against TNBC (NCT03363893) [32]. Most known CDK7 inhibitors are multi-CDK inhibitors that inhibit CDK7 alongside other CDKs (for example, CDK1, CDK2, CDK4/6, CDK9, and CDK12) [6,51,52]. Compared to these, IB has the advantage of providing more specific inhibition of CDK7/9, and there are reports that show it provides more effective inhibition than ribociclib [33]. IB is an isobutyrate ester prodrug of a novel synthetic CDK inhibitor with activity against CDK7 and CDK9 [33]. In a previous study, the kinase assay was performed to prove that IB is a CDK7/9-specific inhibitor. As a result, the values of IC_50_ for IB-mediated inhibition of CDK7 and CDK9 were 0.84 and 0.95 μM, respectively [33].

Additionally, IB can potentially expand the scope of clinical application in breast cancer. Most CDK7 inhibitors are undergoing clinical trials in hormone receptor-positive breast cancer, and the combination of palbociclib (a CDK4/6 inhibitor) and letrozole or tamoxifen (hormone inhibitors) has recently been tried clinically in ER-positive breast cancer [53,54,55,56]. Therefore, we expect IB to have more potential for success in clinical trials.

Herein, we used IB, which is expected to be a new CDK inhibitor in TNBC treatment. First, we investigated the relationship between the OS of patients with TNBC and CDK7/9 expression. These data revealed that patients with high CDK7/9 expression had poor survival rates (Figure 1a). In addition, the survival rate and the incidence of metastasis were higher in patients with high CDK7/9 expression (Figure 1a). Unsurprisingly, IB showed strong cytotoxic effects on TNBC cells (Figure 1b,c). Additionally, we demonstrated that IB-induced cell death was dependent on caspases and that IB decreased the expression levels of anti-apoptotic proteins (Figure 2). Cho et al. [33] reported that IB downregulates anti-apoptosis proteins (Mcl-1, survivin, and XIAP) by inhibiting RNA polymerase II phosphorylation in HCC cells. Based on these reports, we hypothesized that IB may disrupt anti-apoptotic gene transcription regulation, leading to apoptosis in TNBC cells. We further verified the ability of IB to inhibit metastasis of TNBC cells. IB blocked migration and invasion in TNBC cells (Figure 3a,b). However, unlike Hs578T and MDA-MB-231-Luc cells, MDA-MB-435S cells showed fewer metastatic characteristics. Recently, it has been reported that MDA-MB-435S is closer to the melanoma type than breast cancer [57,58]. This could explain the reduced metastatic properties of MDA-MB-435S cells. We evaluated protein level changes of EMT markers to investigate the mechanism underlying the suppression of metastasis by IB. In TNBC cells, MMP-9, MMP-2, and mesenchymal markers (Snail 1/2, TWIST, and ZEB1) were dose-dependently downregulated (Figure 3c,d). Matrix metalloproteinases (MMPs) dissolve the extracellular matrix (ECM) and the basement membrane (BM) to facilitate cancer cell invasion [43]. MMP-2 and MMP-9 play crucial roles in cell migration and early invasion [59]. Although our results showed a decrease in MMP-2 and MMP-9, we conducted additional experiments with only MMP-9. Since MMP-9 expression in TNBC is highly associated with metastasis [60], we hypothesized that MMP-9 is important for suppressing metastasis by IB treatment. To confirm whether MMP-9 is significantly involved in the IB metastasis suppression mechanism, MMP-9 overexpressing cells were established. Migration and invasion assays were performed in MMP-9 overexpressing MDA-MB-231-Luc cells. Therefore, the IB-induced inhibition of metastasis was significantly prevented by MMP-9 overexpression (Figure 3e,f). Collectively, these results indicate that MMP-9 plays a crucial role in IB-induced inhibition of metastasis in TNBC cells. Regrettably, additional experiments were not conducted in the MMP-9 knockdown model (with re-expression of MMP-9) to further emphasize the role of MMP-9 in the inhibition of metastasis by IB. However, since several papers indicate that metastasis is inhibited when MMP-9 is knocked down [61,62,63], IB-induced metastasis inhibition is expected to have a more synergistic effect even in the MMP-9 knockdown model. Although we found that MMP-9 plays an important role in IB-mediated inhibition of metastasis, the mechanism through which IB reduces MMP-9 remains unclear. However, there have been reports that mitogen-activated protein kinase (MAPK)/extracellular signal-regulated kinase (ERK) is involved in metastasis [64,65,66]. Moreover, there are reports that the MAPK/ERK pathway is involved in MMP-9 regulation [67,68,69]. Regrettably, we did not confirm the correlation of MAPK/ERK signaling with MMP-9. To further clarify the mechanism through which IB inhibits metastasis, further studies are needed to investigate the involvement of the MAPK/ERK pathway. 

Furthermore, we evaluated whether IB inhibits metastasis in vivo. Since TNBC spreads to the lungs [47], we constructed an experimental metastasis model that uses an intravenous injection to mimic the spread of MDA-MB-231-Luc cells to the lungs. We designed an experiment in which mice were intravenously injected with MDA-MB-231-Luc cells pretreated with 3 μM IB for 6 h to test whether it could prevent metastasis rather than have an anti-cancer effect on an already metastasized cancer. Using colony-forming assays, we confirmed that tumorigenesis did not differ between untreated and IB-treated cells (Figure S1). Moreover, bioluminescence imaging indicated that mice in the IB-treated group exhibited successful tumor growth inhibition (Figure 4b,c). As previously described in the results (Figure 4b), we observed a phenomenon in which luminescence signals of MDA-MB-231-Luc cells decreased one day after intravenous injection in the experimental metastasis model before subsequently growing again. We confirmed similar trends in some studies that established lung metastasis models with MDA-MB-231-Luc cells [70,71,72]. Due to the size limitation of mouse capillaries, it is almost impossible for human tumor cells to pass through the lungs from the arteries to the venous system [70,73]. Thus, immediately after the tail intravenous injection, all detectable cells become trapped in the lungs. This was why the bioluminescence signal was substantially attenuated in all groups within the first few days. Overall, it has been shown that the bioluminescence signal weakens in the early stages (0–7 days) of the experimental metastasis model; however, it precisely mimics all tumor metastases in the late stages.

In conclusion, this study demonstrated that IB could induce caspase-dependent cell death and suppress metastasis by regulating MMP-9 in TNBC. This preclinical evidence may provide an effective therapeutic strategy against TNBC and a promising clinical candidate as a novel CDK7/9-specific inhibitor.

## 4. Materials and Methods

### 4.1. Cell Culture

The human TNBC cell lines (Hs578T and MDA-MB-435S) were purchased from the American Tissue Culture Collection (ATCC, VA, USA). The human TNBC cell line (MDA-MB-231-Luc) was purchased from Caliper Life Sciences (Hopkinton, MA, USA). Hs578T cells were cultured in DMEM (4.5 g/L d-glucose) (Gibco, Waltham, MA, USA) supplemented with 10% fetal bovine serum (FBS) and 1% penicillin/streptomycin (P/S) (Gibco). MDA-MB-231-Luc cells were maintained in MEM (Gibco) supplemented with 10% FBS and 1% P/S. MDA-MB-435S cells were cultured in DMEM (1 g/L d-glucose) (Gibco) supplemented with 10% FBS and 1% P/S. All cells were incubated in 5% CO_2_ at 37 °C. Cell lines were tested using the MycoAlert PLUS Mycoplasma Detection kit (LT07-710, Lonza, Walkersville, MD, USA). To establish stable cell lines, the MMP-9-FLAG plasmid (RC202872, OriGene, Rockville, MD, USA) was transfected using the FuGENE HD Transfection Reagent (E2312, Promega, Madison, WI, USA) in MDA-MB-231-Luc cells. Transfected cells were selected using 500 μg/mL neomycin (108321-42-2, Corning, Corning, NY, USA).

### 4.2. Reagents

IB was obtained from Seoul National University and dissolved in dimethyl sulfoxide (DMSO) to prepare 10 mM stock solutions for the in vitro experiments. DMSO was purchased from Sigma-Aldrich (St. Louis and Burlington, MA, USA), and z-VAD-fmk from R&D Systems (Minneapolis, MN, USA). The primary antibodies against α-tubulin (2125), cleaved caspase 3 (9661), MMP-2 (4022) were purchased from Cell Signaling Technology (Danvers, MA, USA), β-actin (A5441) from Sigma-Aldrich, MMP-9 (PA5-13199) from Invitrogen (Waltham, MA, USA), Bcl-xL (ADI-AAM-080-E) and Mcl-1 (ADI-AAP-240) from Enzo Life Sciences (Farmingdale, NY, USA), cleaved PARP (ab32561), Snail 1/2 (ab135708), and TWIST (ab175430) from Abcam (Cambridge, UK), survivin (NB500-201) from Novus Biologicals (Centennial, CO, USA), XIAP (610762) from BD Biosciences (Franklin Lakes, NJ, USA), and ZEB1 (polyclonal goat anti-human; sc-10572) from Santa Cruz Biotechnology (Dallas, TX, USA).

### 4.3. Cell Viability and Clonogenic Assays

For cell viability assays, cells were seeded in 24-well plates and treated as indicated in Figure 1b. Cytotoxicity was assayed using CCK-8 (CK04, Dojindo, Mashiki-machi, Tabaru, Japan) according to the manufacturer’s protocol. For clonogenic assays, cells were seeded in 6-well plates and exposed to different doses of IB (1 or 3 μM) for 12 h. The cells were incubated for nine days to allow colony formation and stained with 0.5% crystal violet solution in 10% methanol. Colonies with >50 cells were counted.

### 4.4. Annexin V and PI Staining

Cells were harvested with trypsin and washed with PBS. We performed the experiment using the Annexin-V-FLUOS Staining kit (11-858-777-001, Roche, Basel, Switzerland) according to the provided manual. Cells were stained with 5 μL annexin-V-FLUOS and 1 μL propidium iodide (PI) staining solution in the dark at room temperature for 15 min. The cell samples were analyzed by flow cytometry (FACS Canto II, BD Life Sciences, Franklin Lakes, NJ, USA).

### 4.5. Western Blotting Analysis

Cells were washed in PBS and lysed in a 2x sodium dodecyl sulfate (SDS) sample buffer (ELPIS-BIOTECH, Daejeon, Republic of Korea). The lysates were boiled for 5 min, separated using sodium dodecyl sulfate-polyacrylamide gel electrophoresis (SDS–PAGE), and transferred to an Immobilon membrane. After blocking nonspecific binding sites for 30 min using 5% skimmed milk, membranes were incubated with specific antibodies either at 4 °C overnight or at room temperature for 2 h. Subsequently, membranes were washed three times with TBST and incubated with peroxidase-conjugated donkey anti-rabbit or anti-mouse antibodies at room temperature for 1 h (Jackson ImmunoResearch Laboratories, West Grove, PA, USA). Protein bands were visualized using ECL (Amersham Life Science, Amersham, Bucks, UK) and ImageQuant LAS-4000 (GE Healthcare Life Sciences, Marlborough, MA, USA).

### 4.6. Cell Migration and Invasion Assays

Polycarbonate membrane Transwell with a pore size of 5 μm (3421, Corning) and Matrigel (356237, Corning) was used for the invasion assays. Upper chambers were coated with Matrigel (150 μg/100 μL) and incubated at 37 °C for 30 min before cell seeding. Following Matrigel suction, Hs578T and MDA-MB-231-Luc (3 × 10^4^ cells/100 μL) and MDA-MB-435S (4 × 10^4^ cells/100 μL) cells were seeded into the upper chamber in DMEM (4.5 g or 1 g/L d-glucose). MEM with 10% FBS and 600 μL media was added to the lower chamber. After 24 h at 37 °C with 5% CO_2_ in an incubator, the upper chamber was treated with IB (0.5 and 1 μM) and 0.1% FBS-media for 24 h. The media in the bottom well were replaced with fresh media containing 10% FBS used as a chemoattractant. The non-invasive cells were removed with a cotton swab. After washing with PBS, the upper chamber was fixed, stained with crystal violet (20% methanol + 0.5% crystal violet) for 30 min, and washed. The invasive cells were observed using a microscope (BX53, OLYMPUS, Tokyo, Japan) in five independent fields of view per sample at 4× and 10× magnifications. Wound healing assays were performed to examine cell migration. Cells were seeded (1–3 × 10^4^ cells/70 μL/insert) on Culture-Insert 2 Wells in a μ-dish (81176, ibidi, Gräfelfing, Germany). After 24 h, inserts were removed and scratched. Media, with or without IB (1 and 3 μM), were added, and the cells were incubated for 24 h. Cell migration was monitored using a phase-contrast microscope (IX71, Olympus, Tokyo, Japan) at 10× magnification. ImageJ software was used to evaluate the images.

### 4.7. Gene Expression Omnibus (GEO) Analysis

To complement our experimental results, additional analyses for the association between CDK7/9 gene expression and patient survival were performed using the publicly available GEO database (GSE16446), The Cancer Genome Atlas (TCGA), and the Netherlands Cancer Institute (NKI). Survival estimates were calculated using the Kaplan–Meier method and compared using log-rank tests.

### 4.8. Animal Models

The animal study was approved by the Institutional Animal Care and Use Committee (IACUC) of the Asan Institute for Life Science (2021-12-080).

BALB/c nude mice (6 weeks old, female, SLC, Shizuoka, Japan) were used to generate mouse models. An experimental metastasis model was developed via intravenous injection of MDA-MB-231-Luc cells (1 × 10^6^ cells/100 μL). MDA-MB-231-Luc cells were treated with 3 μM IB 6 h before injection. IB-treated cells were intravenously injected after washing with saline. Tumor growth was monitored through the IVIS spectrum (Perkin Elmer, Waltham, MA, USA).

### 4.9. Statistical Analysis

All data are expressed as the mean ± standard deviation (SD) to represent at least three experiments. Statistical analysis was performed using Student’s *t*-test or one-way analysis of variance (ANOVA) using Prism statistical software (GraphPad Prism 5 Software, La Jolla, CA, USA). *p*-values were obtained using Microsoft Excel 365. Statistical significance was set at *p* < 0.05.

## Data Availability

All data involved in this study are available in the main text or the Supplementary Materials. Materials can be made available through an agreement with the corresponding authors.

## Supplementary Materials

The following supporting information can be downloaded at: https://www.mdpi.com/article/10.3390/ijms25116123/s1.
