# Supplementary material for: Ibulocydine Inhibits Migration and Invasion of TNBC Cells via MMP-9 Regulation
Source: Int J Mol Sci. 2024 Jun 1;25(11):6123. doi: 10.3390/ijms25116123 (PMC11173234; doi:10.3390/ijms25116123)
Supplement: Supplementary file 1 [file ijms-25-06123-s001.zip › ijms-3006281-supplementary.pdf]

Ibulocydine Inhibits Migration and Invasion of TNBC Cells via MMP-9 Regulation

Mi Ri Kwon[1,2,3]†, Ji Soo Park[6]†, Eun Jung Ko[2,3], Jin Park[2,3], Eun Jin Ju[2,3], Seol Hwa Shin[2,3], Ga Won Son[1,2,3], Hye Won Lee[1,2,3], Yun-Yong Park[7], Myoung-Hee Kang[2], Yeon Joo Kim[4], Byeong Moon Kim[6], Hee Jin Lee[8], Tae Won Kim[3,9], Chong Jai Kim[3,8], Si Yeol Song[3,4], Seok Soon Park[2,3]*, Seong-Yun Jeong[1,2,3,5]*.

[1]Department of Medical Science, Asan Medical Institute of Convergence Science and Technology, University of Ulsan College of Medicine, Seoul 05505, Republic of Korea

[2]Asan Institute for Life Sciences, ASAN Medical Center, Seoul 05505, Republic of Korea

[3]Asan Preclinical Evaluation Center for Cancer Therapeutics, ASAN Medical Center, Seoul 05505, Republic of Korea

[4]Department of Radiation Oncology, ASAN Medical Center, University of Ulsan College of Medicine, Seoul 05505, Republic of Korea

[5]Department of Biochemistry and Molecular Biology, ASAN Medical Center, University of Ulsan College of Medicine, Seoul 05505, Republic of Korea

[6]Department of Chemistry, Seoul National University, Seoul 08826, Republic of Korea

[7]Department of Life Science, Chung-Ang University, Seoul 06974, Republic of Korea

[8]Department of Pathology, ASAN Medical Center, University of Ulsan College of Medicine, Seoul 05505, Republic of Korea

[9]Department of Oncology, Asan Medical Center, University of Ulsan College of Medicine, Seoul 05505, Republic of Korea.

*Corresponding author. Email: pssooni@amc.seoul.kr (S.S. Park), syj@amc.seoul.kr (S.-Y. Jeong).

†These authors contributed equally to this work.

Supplementary Figure S1

Original Western blots

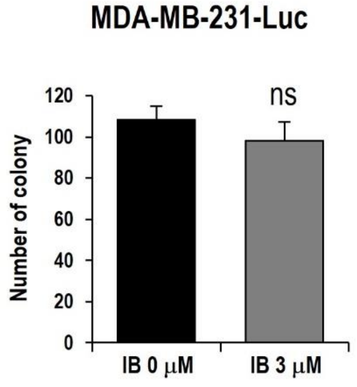

Supplementary Figure S1. Confirmation of the experiments described in Figure 4. Colony formation assays were performed to confirm the absence of differences in tumorigenesis of IB-treated MDA-MB-231-Luc cells for 6 h. Data are shown as the mean ± SD. Abbreviation: ns, non-significant.

Original Western blots

Figure 2A

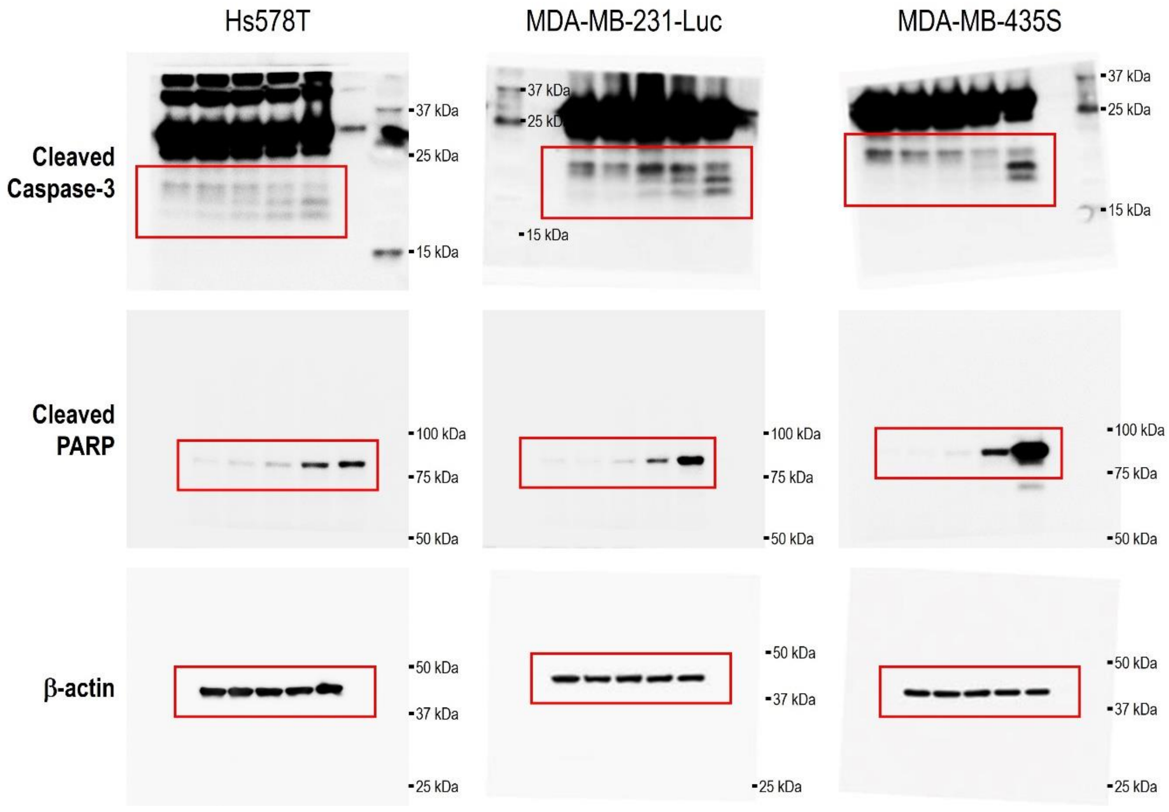

Figure 2B

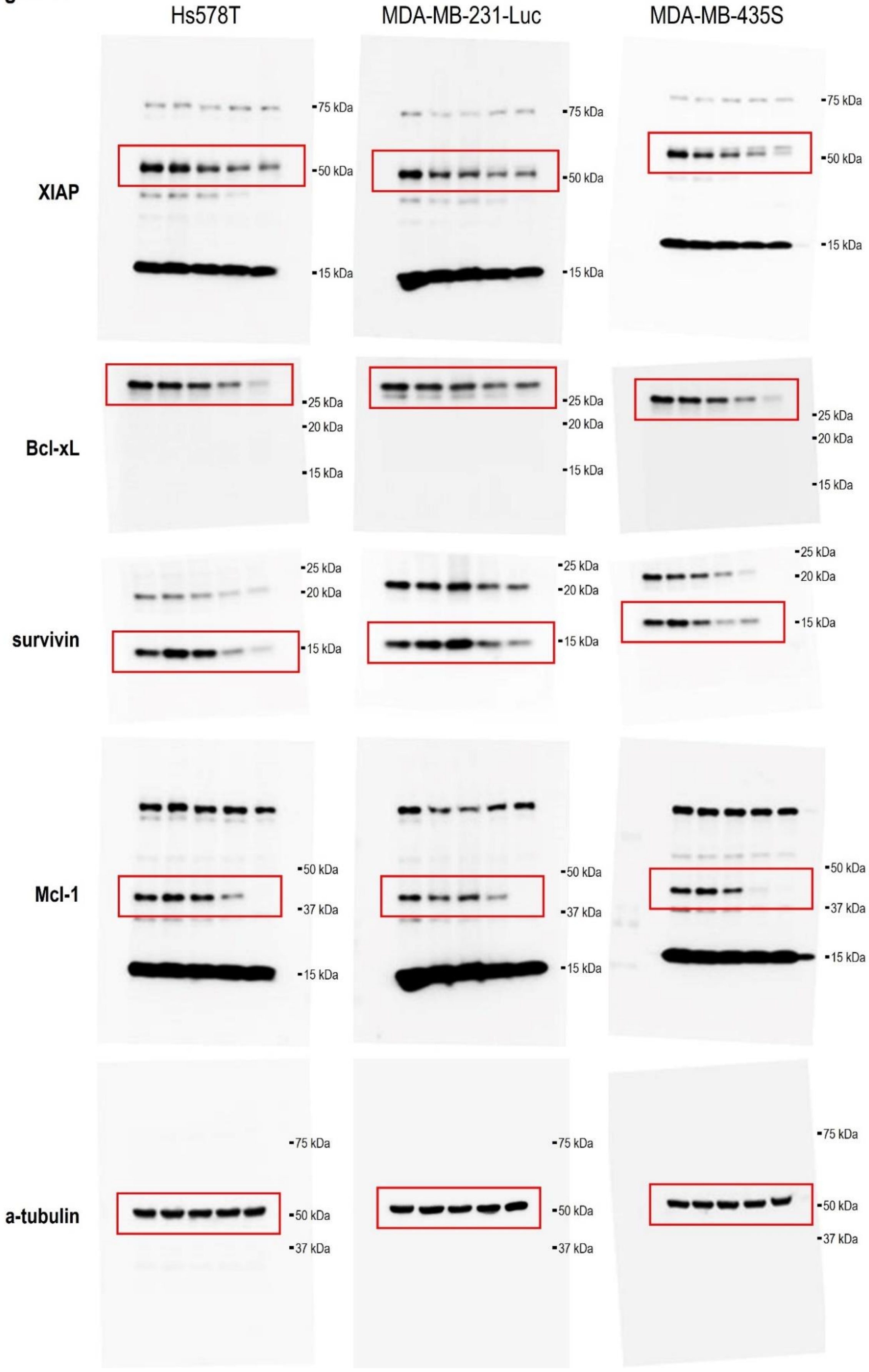
Hs578T
MDA-MB-231-Luc
MDA-MB-435S
XIAP
Bcl-xL
survivin
Mcl-1
a-tubulin
75 kDa
50 kDa
37 kDa
25 kDa
20 kDa
15 kDa

Figure 2D

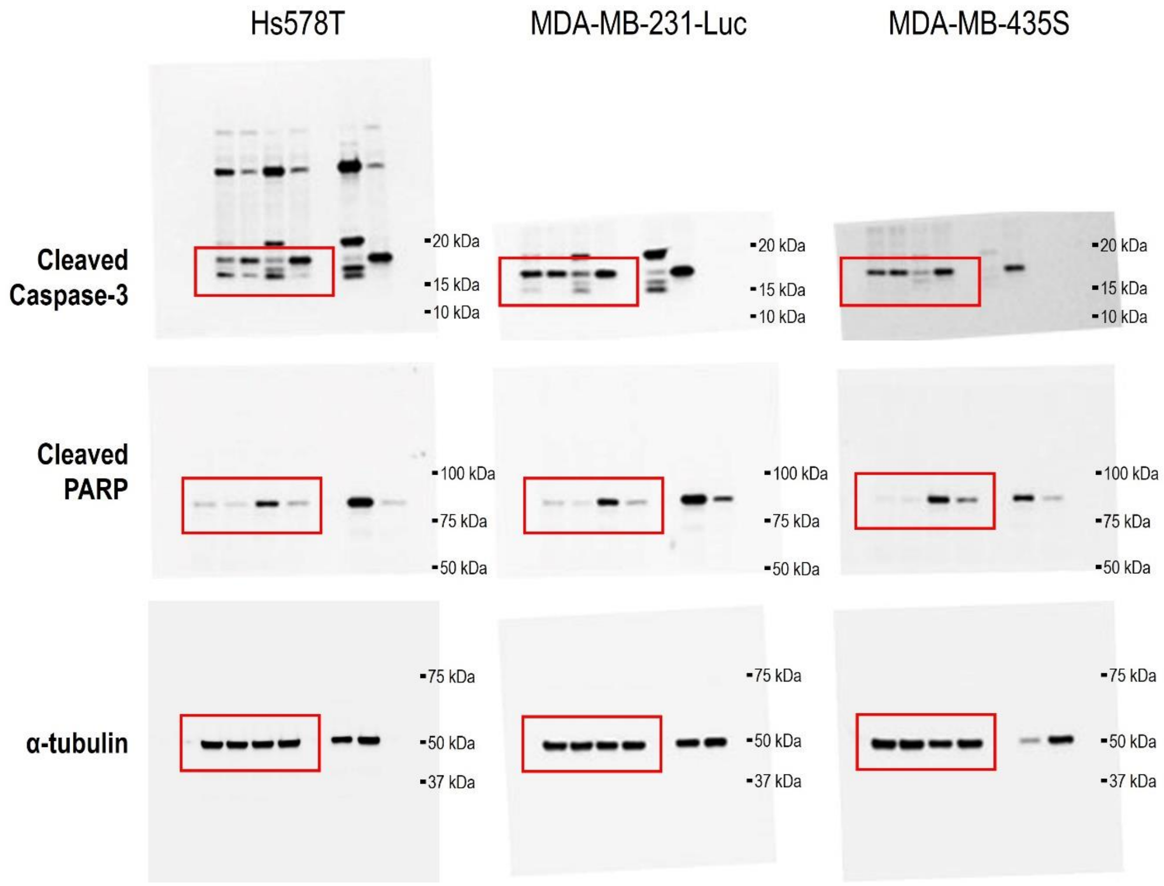
Hs578T
MDA-MB-231-Luc
MDA-MB-435S
Cleaved Caspase-3
Cleaved PARP
α-tubulin
20 kDa
15 kDa
10 kDa
100 kDa
75 kDa
50 kDa
37 kDa

Figure 3C

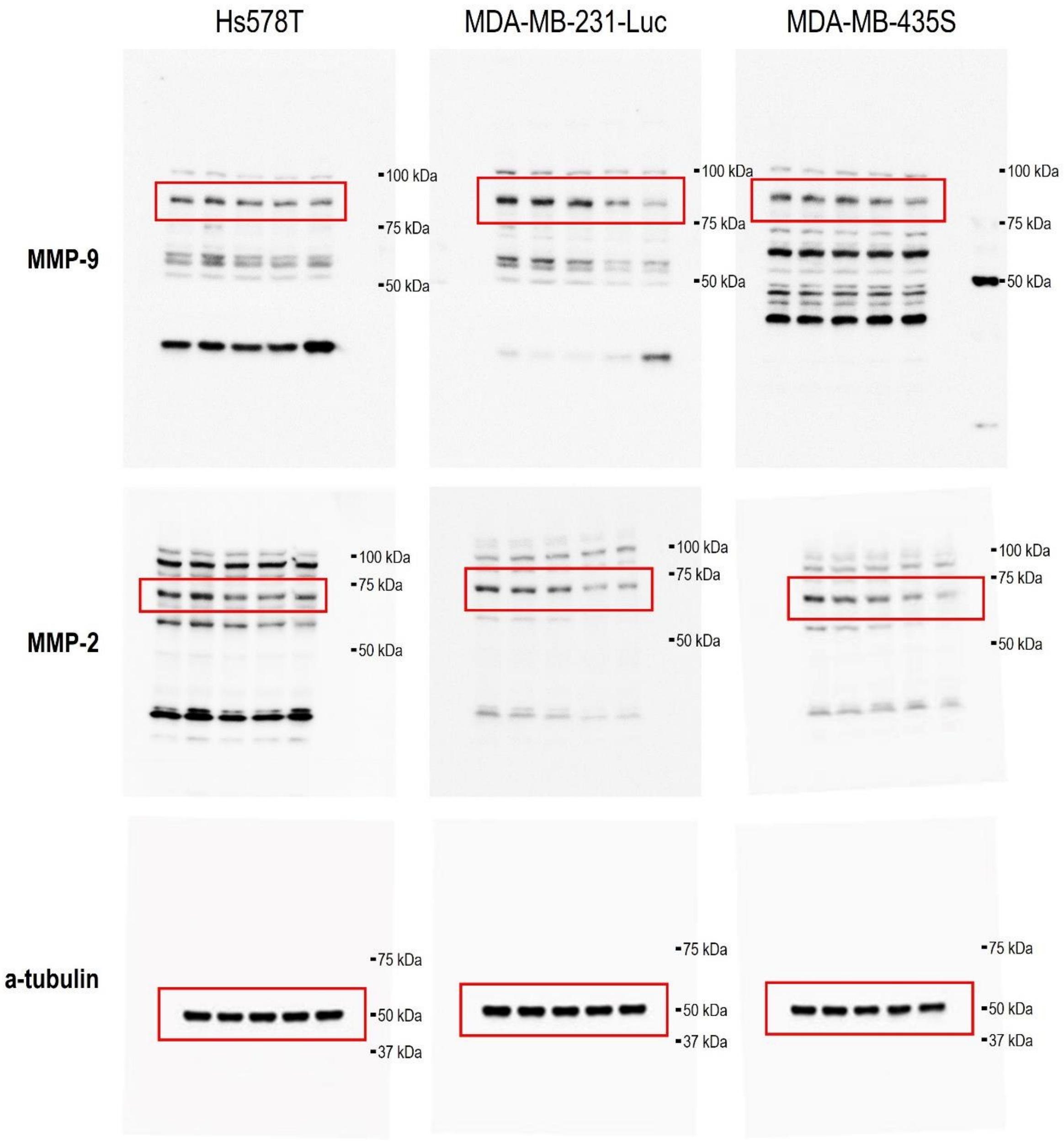
Hs578T
MDA-MB-231-Luc
MDA-MB-435S
MMP-9
MMP-2
a-tubulin
100 kDa
75 kDa
50 kDa
37 kDa

Figure 3D

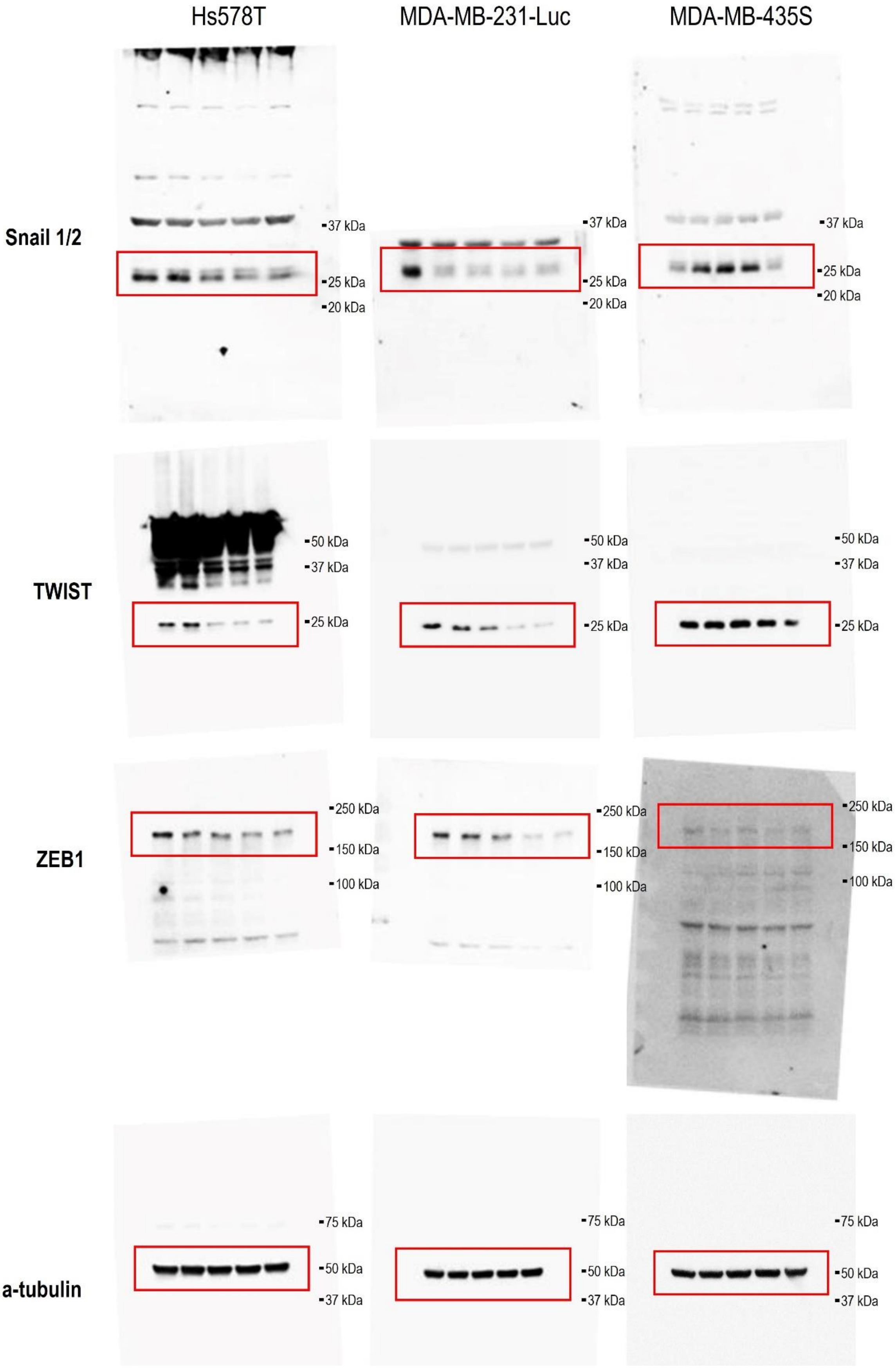
Hs578T
MDA-MB-231-Luc
MDA-MB-435S
Snail 1/2
37 kDa
25 kDa
20 kDa
TWIST
50 kDa
37 kDa
25 kDa
ZEB1
250 kDa
150 kDa
100 kDa
a-tubulin
75 kDa
50 kDa
37 kDa

Figure S1

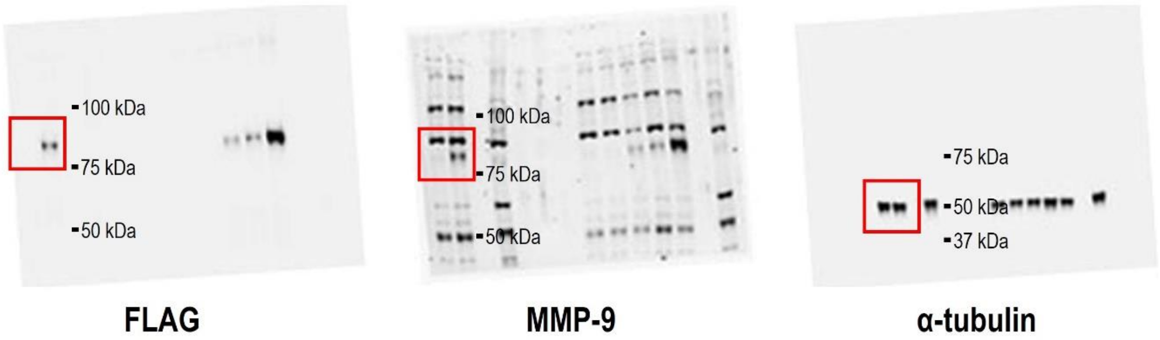

100 kDa
75 kDa
50 kDa
100 kDa
75 kDa
50 kDa
75 kDa
50 kDa
37 kDa
FLAG
MMP-9
α-tubulin
